# The Relationship between Diabetes Family Conflict and Parental Conflict on Problem Recognition in Illness Self-Management among Individuals with Type 1 Diabetes Mellitus

**DOI:** 10.3390/ijerph18178914

**Published:** 2021-08-25

**Authors:** Mi-Kyoung Cho, Mi Young Kim

**Affiliations:** 1 Department of Nursing Science, Chungbuk National University, 1 Chungdae-ro, Seowon-gu, Cheongju 28644, Korea; ciamkcho@gmail.com; 2 College of Nursing, Hanyang University, 222 Wangsimni-ro, Seongdong-gu, Seoul 04763, Korea

**Keywords:** type 1 diabetes, diabetes family conflict, conflict behavior, self-management

## Abstract

We investigated the relationship between diabetes family conflict and parental conflict on problem recognition in illness self-management (PRISM) among individuals with type 1 diabetes mellitus (T1DM). We employed a descriptive research design. Participants were 243 individuals with T1DM who completed online questionnaires. Data were analyzed with descriptive statistics, correlations, and multiple regression analyses. Results revealed that barriers were felt in all areas (understanding and organizing care, regimen pain and bother, healthcare team interaction, family interaction, and peer interaction), especially peer interaction. The significant influencing factors in the regression model for the total PRISM score of individuals with T1DM were conflict behavior toward mothers (t = 4.44, *p* < 0.001), diabetes family conflict (t = 5.77, *p* < 0.001), conflict behavior toward fathers (t = 2.58, *p* = 0.011), women (t = 2.67, *p* = 0.008), non-religious (t = −2.33, *p* = 0.020), and diabetic complications (t = 2.17, *p* = 0.031). The explanatory power of the constructed regression model for PRISM was 42.0% (F = 30.12, *p* < 0.001). To promote self-management among individuals with T1DM, the development of interventions that promote improved peer interactions, a family-centered approach, and a program that can minimize conflicts between families and parents are required.

## 1. Introduction

Type 1 diabetes mellitus (T1DM) is an autoimmune disease in which insulin deficiency is caused by the destruction of beta cells [1]. T1DM is one of the most common chronic diseases, with approximately 70,000 children diagnosed with it each year worldwide [2]. The Asian population has a relatively lower incidence than the Caucasian population [2]. However, the incidence rate in children and adolescents in Korea has increased from 32.85 per 100,000 persons in 2007 to 41.03 per 100,000 persons in 2017 [3]. Therefore, it is necessary to pay attention to T1DM, which requires lifelong disease management, and the problems caused by the disease.

T1DM requires continuous adaptation and management, and persons should start effective self-management through self-management education from the time of the first diagnosis [4]. Self-management of T1DM is a broad field that includes insulin administration, blood glucose monitoring, adherence to a regular diet, exercise, and decision-making on disease management [5]. Self-management for T1DM is quite complex owing to the nature of the disease. To maintain proper blood sugar levels, children with T1DM and their parents must cope with a complex and demanding daily treatment regime, including blood glucose monitoring (several times a day), administering insulin correctly and accurately, regulating food intake, and monitoring physical activity [6]. When diabetes is poorly managed, complications can lead to severe morbidity or mortality [7]. Diabetes-related complications include small vascular complications in the retinal peripheral nerve and kidneys that can lead to retinopathy/neuropathy and nephropathy disease, respectively; additionally macrovascular complications including peripheral artery disease, coronary artery disease, and cerebrovascular disease [7].

The problems that arise when self-management is not performed properly are not only health-related but also psychosocial, such as the stress caused by difficulties in management itself, which further negatively affects diabetes outcomes including quality of life and blood sugar control [8]. As the importance of self-management in T1DM has risen, interest in self-management is increasing [9], and most studies have viewed self-management as a single domain or focused on family interactions or on a subgroup of factors such as psychosocial barriers [10,11]. However, as self-management is complex, it is important to divide it into sub-categories and consider which parts are difficult.

Regarding the self-management of T1DM, Cox [12] divided self-management barriers into understanding and organizing care, regimen pain and bother, healthcare team interactions, family interactions, and peer interactions. We followed Cox’s classification in this study to determine the factors influencing self-management barriers.

Concerning family-related factors, just as it is necessary to consider the culture of individuals with diabetes to promote effective self-management of diabetes, it is important to consider the influence of the family and the influence of family conflicts owing to the characteristics of Korean culture [13]. In managing children’s chronic health problems, various socio-economic difficulties arise in families, and the resulting emotional pressure increases family stress and can negatively affect family relationships. In addition, children’s chronic diseases can cause changes in or overburdening of the roles played by family members, affect interactions among family members, cause long-term tension, and lead to imbalances in the daily family lifestyle. It can affect the lives of individual family members as well as impair normal family functioning [14]. T1DM is relevant to all family members because it requires a change in overall lifestyle. Since family conflict is likely to increase in the presence of T1DM [15] and the cultural characteristics of Korea include valuing family, the degree of influence of diabetes family conflict (DFC) on the self-management of diabetes in individuals with T1DM was explored in this study.

Concerning the relationship between parents and children, unlike the Western family culture that emphasizes the independence and autonomy of children, Korea has a family culture that values the interdependence of parents and children [16]. Concerning treatment of T1DM, parents and children may have conflicts over blood sugar management [17,18]. In fact, when undergoing continuous glucose monitoring, which is useful in many aspects of blood sugar management, parents expressed that continuous glucose monitoring reduced the stress on blood sugar management; whereas in the case of their children, depending on the blood sugar level measured, being asked “what they had done wrong” made them feel like they were being “spied” on [18]. Even devices to help manage blood sugar can cause conflicts with parents in the process of managing blood sugar. Parents may talk to their children in the hopes of helping them appropriately manage their blood sugar, but this may become a factor that makes individuals with T1DM unable to adequately manage their blood sugar. In fact, a previous study found that family conflict had a negative effect on blood sugar management [19]. In addition, it was reported that controlling blood sugar in children becomes difficult if the communication skills between parents and children are insufficient [20]. Therefore, interventions to relieve conflicts with parents should be given priority. The significance of this study could be lies in how it identifies areas where self-management is difficult. The results of this study indicate that basic data can be prepared for developing an effective intervention program with limited resources. In addition, this study is important because the method employed considers the participants’ family situation.

Therefore, we examined the problem recognition in illness self-management (PRISM), DFC, and parental conflict among individuals with T1DM, including the effect on PRISM. The research questions of this study were as follows.

(1) What is the level of PRISM, DFC, and conflict with parents of individuals with T1DM?

(2) What is the difference in PRISM according to the characteristics of individuals with T1DM?

(3) What factors affect the PRISM of individuals with T1DM?

## 2. Materials and Methods

### 2.1. Research Design

This study employed a descriptive research design to examine the PRISM, DFC, conflict behavior toward mother (CBM), and conflict behavior toward father (CBF) of individuals with T1DM and to identify the factors influencing PRISM.

### 2.2. Participants

Participants were aged ≥ 10 years, self-managing diabetes, and could participate in the survey independently. Participants understood the purpose of the study and agreed to participate. Those who were participating in an intervention program of self-management for T1DM during the data collection period and those with cognitive or psychiatric disorders were excluded. The number of participants required (*n* = 178) was calculated using a two-sided test with a significance level (α) of 0.05, median effect size (f2) of 0.15 [21], power (1-β) of 0.95, and the number of predictors (*n* = 11) using G*power 3.1.2 [22]. Considering a 25% dropout rate, the target rate was 238 participants. Finally, data from 243 participants were analyzed (the final power of this study was 1.00).

### 2.3. Research Tools

#### 2.3.1. Participant Characteristics

We investigated the sociodemographic and disease-related characteristics of individuals with T1DM, including sex, religion, occupation, diabetes education, diabetic complications, disease duration, and glycosylated hemoglobin (HbA1C) values recently measured at a hospital.

#### 2.3.2. Problem Recognition in Illness Self-Management (PRISM)

PRISM refers to identifying the unique barriers experienced by T1DM patients and their parents [12]. The PRISM scale used in this study was developed for T1DM participants by Cox et al. [12]. When measuring adolescent parents, all six sub-domains and 28 items were measured; however, when targeting diabetes patients, 21 questions under only five out of six PRISM sub-domains (understanding and organizing care, regimen pain and bother, healthcare team interaction, family interaction, and peer interaction) were measured. Scores range from 1 (“not at all”) to 5 (“very much so”) on a 5-point Likert-type scale. Five items are reverse-scored and converted; higher total scores and higher scores in each sub-domain indicate greater barriers to self-management experienced by individuals with T1DM. The average score for each sub-area is calculated by dividing the sum of the responses to the questions related to the barriers in each sub-area by the number of questions in the corresponding barrier. When the average score exceeds 2, it identifies a barrier, and a higher score means that it can have a greater effect on the glycemic control of persons with T1DM. In this study, Cronbach’s α was 0.878, indicating good reliability.

#### 2.3.3. Diabetes Family Conflict (DFC) Scale

DFC is a conflict between a child with T1DM and a parent, and the burden and demand for treatment therapy appear as a conflict between the parent and child [23]. In this study, a tool developed by Rubin et al. [24] and updated by Hood et al. [25] was used, which comprises 19 items concerning family conflicts related to diabetes management, such as insulin application, blood glucose measurement, and talking to others about diabetes. Each item is rated on a 3-point scale (1 = never, 2 = sometimes, or 3 = always). Scores range from 19 to 57, with higher scores indicating greater conflict. Cronbach’s α in Hood et al.’s study was 0.85; in this study, Cronbach’s α was 0.937, indicating excellent reliability.

#### 2.3.4. Conflict Behavior Questionnaire (CBQ)

Conflict behavior is defined as a clash between individuals arising out of a difference in thought processes, attitudes, understanding, interests, requirements, or perceptions [26]. The CBQ used in this study was developed by Robin and Foster [27], modified by Holmes and colleagues [28] (CBQ-20), translated into Korean by Jang and Park [29], and validated. The CBQ describes dissatisfaction in parent–child relationships as perceived by adolescents such as lack of dialogue, disagreement, and closed communication. Eight negative questions were reverse-coded. Responses were measured with a 5-point Likert-type scale: 1 (“not at all”) to 5 (“strongly agree”). In Jang and Park [29], Cronbach’s αs were 0.95 for CBF and 0.96 for CBM. In this study, Cronbach’s αs were 0.950 for CBF and 0.956 for CBM, indicating excellent reliability.

### 2.4. Research Ethics

This study was approved by the Research Ethics Committee (No. EU17-41). There was no risk to the participants as an online survey was used; however, since the participants had T1DM, we made every effort to protect their voluntary participation and personal information.

All participants signed an informed consent form, which included a description of the study. The participants were informed of the purpose and procedure of the study, as well as of their rights, and they were guaranteed confidentiality. Additionally, when the participants accessed the survey URL, they read the research description and the online consent form for the participants on the first screen; if they selected “I agree” on the online consent form, they were redirected to the survey screen. By doing so, only those individuals who voluntarily consented to participate in the study after reading the online consent form were allowed to complete the survey. Participants who were children (aged < 18 years) were asked to participate in the survey only if a guardian’s or parent’s consent was provided.

### 2.5. Data Collection Method

Data collection was conducted through an online survey from 5–29 August 2019. The purpose and procedure of the study were notified after obtaining permission from the online community executive. The survey URL home screen provided explanations concerning the research purpose, survey method, and rights and personal information protection of research participants. Only those who read the research description before starting the survey and agreed to participate in the survey were allowed to voluntarily participate. Data concerning PRISM, DFC, CBM, and CBF were collected.

### 2.6. Data Analysis Method

Data were analyzed using the IBM SPSS Statistics 25.0 program (Chicago, IL, USA).

Participants’ characteristics, PRISM, DFC, CBM, and CBF were analyzed with mean, standard deviation, frequency, and percentage. The differences in PRISM according to the characteristics of individuals with T1DM was analyzed with independent *t*-tests; and the correlations between PRISM, DFC, CBM, and CBF were analyzed with Pearson’s coefficient correlations. The associations between the characteristics of individuals with T1DM, DFC, CBM, and CBF on PRISM were analyzed with stepwise multiple regression analyses. Significance was set at *p* < 0.05.

## 3. Results

### 3.1. Participants’ Characteristics

The average age of the study participants was 26.71 ± 11.48 years and 63 patients (25.9%) were aged below 18 years; by sex, 159 (65.4%) were female, 158 (65.0%) were non-religious, 204 (84.0%) had a job, 230 (94.7%) had diabetes education. and 53 (21.8%) had diabetes complications. The average duration of the disease was 10.44 ± 8.04 years, and 148 participants (60.9%) were below the average; the average HbA1C was 7.12 ± 1.39% and 85 participants (35.0%) were above the average (Table 1).

### 3.2. PRISM, DFC and CBQ

The average total score of PRISM was 53.90 ± 13.09 (range: 24–89), and when converted to a score of 5, the average score was 2.57 ± 0.62 (range: 1.14–4.24), exceeding 2 points, indicating that the overall self-management barrier was high. For each sub-domain of PRISM, peer interaction had an average score of 3.25 ± 1.11, regimen pain and bother had an average score of 2.72 ± 0.90, healthcare team interaction had an average score of 2.54 ± 0.78, family interaction had an average score of 2.38 ± 0.81, and understanding and organizing care had an average score of 2.21 ± 0.86, where all areas exceeded 2 points, indicating that the barrier to self-management was high, and among them, peer interaction was the highest (Table 2). Mean DFC was 26.60 ± 7.25 (range: 19–51), mean CBF was 44.75 ± 15.95 and that of CBM was 39.44 ± 15.49 (range: 20 to 100).

### 3.3. Differences in PRISM According to Participants’ Characteristics

PRISM was higher in participants with no religion, diabetic complications, and those with and HbA1C value of 7.12% or greater as compared to their counterparts. In the PRISM sub-area “understanding and organizing care,” self-management barriers were high in non-religious participants and participants with an HbA1C value of 7.12% or greater; meanwhile, “regimen pain and bother” was high in those who were aged above 18 years, non-religious, and had an HbA1C value of 7.12% or greater. In the “healthcare team interaction” sub-area, the barriers to self-management were high in those who were aged above 18 years, non-religious, unemployed, had complications, and had an HbA1C of 7.12% or higher. In the “family interaction” sub-area, the barriers to self-management were high in those who were aged above 18 years, had complications, and had an HbA1C of 7.12% or higher. Lastly, in the “peer interaction” sub-area, self-management barriers were high in those with complications (Table 3).

### 3.4. Correlations between PRISM, DFC and CBQ

Among the PRISM total scores and sub-domains, understanding and organizing care, regimen pain and bother, healthcare team interaction, and family interaction were significantly positively correlated with DFC, CBM, CBF, and HbA1C (Table 4). Peer interaction was significantly positively correlated only with DFC and CBM.

### 3.5. Factors Influencing PRISM

Table 5 shows the regression model to check the factors influencing PRISM and its sub-domains among individuals with T1DM. Categorical variables such as sex, religion, occupation, diabetes education, and diabetic complications were treated as dummy variables; and age, illness duration, HbA1C, DFC, CBF, and CBM were entered as continuous variables and analyzed using stepwise multiple regression analyses. When constructing the model, variables were selected based on a significant probability of 0.05, and variables were removed based on a significant probability of 0.10. In the PRISM model, the tolerance limits between independent variables were all higher than 0.1, the standard; and the variance expansion index (VIF) also satisfied the standard value of 10 or less, indicating that there was no problem of multicollinearity.

The significant influencing factors in the regression model for the total PRISM score of individuals with T1DM were CBM, DFC, CBF, women, non-religious, and diabetic complications. The explanatory power of the regression model constructed with these six variables for PRISM was 42.0% (F = 30.12, *p* < 0.001).

By sub-domain of PRISM of individuals with T1DM, the significant influencing factors in the regression model for understanding and organizing care were CBM, DFC, and non-religious. The explanatory power of the barrier to understanding and organizing care of the regression model constructed with these three variables was 29.6% (F = 34.75, *p* < 0.001). The significant influencing factors in the regression models for regimen pain and bother were DFC, CBF, women, and non-religious. The explanatory power of the barrier to regimen pain and bother of the regression model constructed with these four variables was 25.0% (F = 21.13, *p* < 0.001). In the regression model for healthcare team interaction, the significant influencing factors were CBM and illness duration. The explanatory power of the barrier to the healthcare team interaction of the model was 12.8% (F = 18.77, *p* < 0.001). The significant influencing factors in the regression model for family interaction were CBM, CBF, and diabetic complications. The explanatory power of the barrier to family interaction of the regression model constructed with these three variables was 44.8% (F = 66.21, *p* < 0.001). The significant influencing factors in the regression model for peer interaction were CBM, DFC, women, and diabetic complications. The explanatory power of the barrier to peer interaction of the regression model constructed with these four variables was 10.5% (F = 8.09, *p* < 0.001).

## 4. Discussion

This study examined the degree of conflict between PRISM and DFC among participants with T1DM and their parents, and we identified the factors influencing PRISM among participants with T1DM. Participants with T1DM felt barriers to self-management in all areas of self-management: understanding and organizing care, regimen pain and bother, healthcare team interaction, family interaction, and peer interaction, which was consistent with a prior study [30]. The difficulties with self-management may be because T1DM requires management in various areas such as insulin injection, blood sugar monitoring, diet, and exercise [31], which means a wide range of characteristics need to be managed. Barriers to self-management do not end only with poor management. As stress from self-management of diabetes negatively affects diabetes outcomes, including quality of life, self-management, and glycemic control [8,32], management barriers are related to physical and psychological aspects.

Among the areas where barriers were felt, the most reported was peer interaction. Considering that peer interactions encompass the importance of peers and beliefs about how peers will react to youths’ illness [12], it is clear that the reactions and support of friends, colleagues, and teachers are important. This is consistent with the reports that individuals with T1DM will eventually take actions other than those they should do for disease control owing to being conscious of the public’s negative perception of needles and how their actions will be seen in a situation where they have to administer drugs in public [33]. Consequently, there is a social stigma associated with diabetes, which appears in both children and adults, and can be characterized by accusations, negative social judgments, stereotypes, exclusion, rejection, and discrimination [34]. Therefore, it was found that not only difficulties caused by the disease, but also suffering felt in peer groups and society as well as experiences in society, have a great influence on disease management.

Second, pain and bother (i.e., feelings about the positive and negative aspects of the self-management regimen [12]) means that individuals complain more about emotional difficulties rather than disease or management itself. With T1DM, the rate of occurrence of psychosocial problems such as irritation, depression, and anxiety was 55.95%, which supports the results of a prior study [35]. This means that psychosocial difficulties related to disease management are ultimately related to actual disease management. Assessment is required to address negative feelings about disease management.

In the regression model for the self-management barriers of the individuals with T1DM, DFC, CBF, CBM, women, non-religious, and diabetic complications were significant influencing factors. This significant influencing factor explained 42.0% of the model. When the factors associated with self-management barriers were divided by area, the factors that had a common influence were DFC, CBM, and CBF.

First, the fact that family conflict related to diabetes is a significant influencing factor on the barrier to self-management is consistent with the fact that when family function is reduced owing to family conflict, it negatively affects the adaptation to the disease of individuals with T1DM [36] and that they find it more difficult to manage glycemic levels [19]. In addition, family conflicts related to diabetes result in poorer metabolic control and are related to reports of higher psychological distress [37]. Accordant with the statement that chronic stress caused by treatment management of T1DM can increase family conflict [38], families coping with T1DM are vulnerable to family conflict. The present results—that this family conflict creates a barrier to self-management of diabetes—suggest the importance of assessing and managing family conflict in those coping with T1DM. According to the Family Management Style Framework related to chronic diseases reported overseas, the definition of family members’ situation incorporates the condition of children with chronic diseases, the management behavior practiced for managing the chronic disease, and the awareness and specific management behavior of the child’s condition. The family management style was identified based on the effect of the three sociocultural context categories. Previous studies suggest that the focus should be on whether children’s disease management is performed as part of their family’s daily lives [39]. Thus, family-centered interventions are important for T1DM management.

Conflict with parents was a factor influencing the barriers to self-management in T1DM. The result that the relationship with parents influences children’s disease management is related to the result that parents’ parenting style influenced their children’s disease management [20]. Children’s adherence to treatment was high in the case of an authoritative parenting presence with mild and structured characteristics; whereas, when communication skills were lacking, children’s adherence to treatment was low, and they had problems with blood sugar control [20]. Parental interventions related to blood sugar management (for example, asking why children did not check their blood sugar in advance, why they did not bring a snack, and how they reacted when their blood sugar level was low) lead to the feeling of being criticized [34], which suggests that interventions for better disease management may have the opposite effect. Additionally, higher self-management barriers in the absence of religion indicated that psychosocial aspects affected by religion also influence the self-management barriers, as has been reported in a study on chronic disease that showed higher psychosocial growth in the presence of religion [40].

Disease duration and HbA1C were non-significant influencing factors affecting the barriers to T1DM self-management. This result suggests that the disease-related characteristics themselves do not affect the self-management barrier of the disease, but rather are related to the influence of surrounding resources or to accepting and coping with the disease. When patients with chronic diseases accept the disease, pain, depression, and anxiety decrease, and physical well-being and quality of life improve [41]. Moreover, self-management behavior increases [42]. These are all consistent with the current results.

A family-centered approach is necessary for families coping with T1DM [32]. Moreover, relationships with peers and ongoing emotional support are also important [43]. Thus, we propose camps to foster exchanges between peers, the development of peer support programs, self-help groups, and peer relations promotion programs such as peer relations interventions in schools.

This study had some limitations. First, in addition to the variables measured in this study, it is necessary to consider other variables, such as social or peer support, that may affect self-management barriers. Second, our examination of the correlations between the factors hinders our ability to infer causality. Third, since this study only targeted those with T1DM, we propose a comparative analysis by expanding the scope to families. Finally, we employed a cross-sectional design; therefore, future researchers may wish to utilize a more in-depth longitudinal design.

## 5. Conclusions

This study was conducted to examine the degree of conflict between PRISM and DFC among participants with T1DM and their parents, and to identify factors affecting PRISM of individuals with T1DM. All five areas were associated with difficulties in self-management, with peer interaction being the highest barrier. Peer support is important for this, and it is necessary to produce a way to improve peer interactions. In addition, conflicts between DFC and parents were common factors affecting self-management. Therefore, to improve self-management of T1DM, it is necessary to develop a family-centered approach and minimize conflicts between families and parents.

In conclusion, self-management resources should be tailored to specific and identifiable self-management barriers. T1DM is a disease in which self-management is vital, and this study is meaningful in that it identified the areas where self-management is difficult. The data inform the development of an effective intervention program. Therefore, through this study, basic data for conducting research in consideration of family and social support aspects when managing chronic diseases as well as type 1 diabetes were presented.

## Figures and Tables

**Table 1:** Participants’ characteristics (*n* = 243).

Characteristic		*n*	%	M ± SD
Age (years)	<18	63	25.9	26.71 ± 11.48
	≥18	180	74.1	
Sex	Male	84	34.6	
	Female	159	65.4	
Religion	No	158	65.0	
	Yes	85	35.0	
Job	No	39	16.0	
	Yes	204	84.0	
Education	No	13	5.3	
	Yes	230	94.7	
Complications	No	190	78.2	
	Yes	53	21.8	
Disease duration (years)	<10.44	148	60.9	10.44 ± 8.04
	≥10.44	95	39.1	
HbA1C (%)	<7.12	157	64.6	7.12 ± 1.39
	≥7.12	85	35.0	

Note. *n*: number, M: mean, SD: standard deviation, HbA1C: glycosylated hemoglobin.

**Table 2:** Descriptive statistics of the examined variables (*n* = 243).

Variables	Items	Scores		5-Point Scale Conversion Scores		Barrier Recognition	
		M ± SD	Min–Max	M ± SD	Min–Max	No	Yes
PRISM	21	53.90 ± 13.09	24–89	2.57 ± 0.62	1.14–4.24		
Understanding and organizing care	5	11.07 ± 4.32	5–22	2.21 ± 0.86	1–4	122 (50.2)	121 (49.8)
Regimen pain and bother	4	10.89 ± 3.61	4–20	2.72 ± 0.90	1–5	66 (27.2)	177 (72.8)
Healthcare team interaction	5	12.69 ± 3.90	5–25	2.54 ± 0.78	1–5	76 (31.3)	167 (68.7)
Family interaction	4	9.51 ± 3.25	4–19	2.38 ± 0.81	1–5	97 (39.9)	146 (60.1)
Peer interaction	3	9.74 ± 3.33	3–15	3.25 ± 1.11	1–5	47 (19.3)	196 (80.7)
DFC	19	26.60 ± 7.25	19–51				
CBF	20	44.75 ± 15.95	20–100	2.24 ± 0.80	1–5		
CBM	20	39.44 ± 15.49	20–100	1.97 ± 0.77	1–5		

Note. *n*: number, M: mean, SD: standard deviation, PRISM: Problem Recognition in Illness Self-Management, DFCS: Diabetes Family Conflict, CBF: conflict behavior toward father, CBM: conflict behavior toward mother.

**Table 3:** PRISM scores by participants’ characteristics (*n* = 243).

Characteristics		PRISM		PRISM Subgroup									
				Understanding and Organizing Care		Regimen Pain and Bother		Healthcare Team Interaction		Family Interaction		Peer Interaction	
		M (SD)	t (*p*)	M (SD)	t (*p*)	M (SD)	t (*p*)	M (SD)	t (*p*)	M (SD)	t (*p*)	M (SD)	t (*p*)
Age (years)	<18	51.14 (13.92)	−1.95 (0.052)	11.30 (4.73)	0.47 (0.637)	10.05 (3.83)	−2.18 (0.030)	11.48 (4.21)	−2.91 (0.004)	8.60 (2.95)	−2.61 (0.010)	9.71 (3.26)	−0.08 (0.933)
	≥18	54.87 (12.69)		10.98 (4.17)		11.19 (3.49)		13.11 (3.70)		9.83 (3.29)		9.76 (3.36)	
Sex	Male	52.25 (12.50)	1.43 (0.153)	10.77 (4.57)	0.77 (0.445)	10.49 (3.50)	1.27 (0.204)	12.31 (3.61)	1.10 (0.273)	9.49 (2.98)	0.08 (0.938)	9.19 (3.33)	1.90 (0.059)
	Female	54.77 (13.35)		11.22 (4.19)		11.11 (3.66)		12.89 (4.04)		9.52 (3.39)		10.04 (3.30)	
Religion	No	56.20 (12.70)	3.84 (<0.001)	11.80 (4.41)	3.86 (<0.001)	11.45 (3.56)	3.35 (0.001)	13.18 (3.71)	2.71 (0.007)	9.79 (3.09)	1.85 (0.066)	9.99 (3.24)	1.55 (0.122)
	Yes	49.62 (12.80)		9.71 (3.82)		9.86 (3.48)		11.78 (4.09)		8.99 (3.47)		9.29 (3.45)	
Job	No	55.97 (12.48)	1.08 (0.281)	11.31 (4.01)	0.38 (0.704)	11.00 (3.48)	0.20 (0.840)	14.13 (3.81)	2.55 (0.011)	9.85 (3.57)	0.70 (0.482)	9.69 (3.59)	0.11 (915)
	Yes	53.50 (13.20)		11.02 (4.38)		10.87 (3.64)		12.41 (3.86)		9.45 (3.19)		9.75 (3.29)	
Education	No	59.08 (10.56)	1.47 (0.143)	12.92 (4.82)	1.60 (0.111)	11.38 (4.07)	0.50 (0.615)	14.08 (2.33)	2.11 (0.051)	10.38 (2.36)	1.00 (0.319)	10.31 (2.56)	0.63 (0.532)
	Yes	53.61 (13.18)		10.96 (4.28)		10.87 (3.59)		12.61 (3.96)		9.46 (3.29)		9.71 (3.37)	
Complications	No	52.64 (13.01)	2.89 (0.004)	10.91 (4.39)	1.10 (0.273)	10.76 (3.63)	1.06 (0.289)	12.37 (3.90)	2.40 (0.017)	9.14 (3.08)	3.47 (0.001)	9.46 (3.17)	2.37 (0.021)
	Yes	58.43 (12.49)		11.64 (4.06)		11.36 (3.53)		13.81 (3.72)		10.85 (3.51)		10.77 (3.69)	
Disease duration (year)	<10.44	53.64 (13.15)	0.38 (0.701)	11.11 (4.64)	0.19 (0.849)	11.05 (3.76)	0.83 (0.406)	12.36 (3.73)	1.65 (0.100)	9.41 (3.08)	0.63 (0.531)	9.72 (3.26)	0.13 (0.899)
	≥10.44	54.31 (13.06)		11.00 (3.79)		10.65 (3.37)		13.20 (4.12)		9.67 (3.50)		9.78 (3.44)	
HbA1C (%)	<7.12	51.50 (12.36)	4.04 (<0.001)	10.35 (3.85)	3.47 (0.001)	10.32 (3.51)	3.45 (0.001)	12.20 (3.86)	2.70 (0.007)	9.01 (3.13)	3.25 (0.001)	9.62 (3.29)	0.87 (0.388)
	≥7.12	58.42 (13.34)		12.45 (4.80)		11.96 (3.57)		13.60 (3.85)		10.40 (3.29)		10.01 (3.40)	

Note. *n*: number, M: mean, SD: standard deviation, HbA1C: glycosylated hemoglobin. PRISM: Problem Recognition in Illness Self-Management.

**Table 4:** Correlations between the examined variables (*n* = 243).

Variables	PRISM	PRISM Subgroup				
		Understanding and Organizing Care	Regimen Pain and Bother	Healthcare Team Interaction	Family Interaction	Peer Interaction
	r (*p*)					
PRISM	1					
DFCS	0.50 (<0.001)	0.49 (<0.001)	0.44 (<0.001)	0.22 (.001)	0.35 (<0.001)	0.25 (<0.001)
CBF	0.37 (<0.001)	0.24 (<0.001)	0.28 (<0.001)	0.24 (<0.001)	0.50 (<0.001)	0.09 (0.150)
CBM	0.54 (<0.001)	0.42 (<0.001)	0.36 (<0.001)	0.28 (<0.001)	0.64 (<0.001)	0.24 (<0.001)
HbA1C	0.27 (<0.001)	0.21 (0.001)	0.25 (<0.001)	0.18 (0.006)	0.23 (<0.001)	0.08 (0.217)

Note. PRISM: Problem Recognition in Illness Self-Management, DFCS: Diabetes Family Conflict, CBF: conflict behavior toward father, CBM: conflict behavior toward mother, HbA1C: glycosylated hemoglobin.

**Table 5:** Influencing factors on PRISM (*n* = 243).

Variables	PRISM		PRISM Subgroup									
			Understanding and Organizing Care		Regimen Pain and Bother		Healthcare Team Interaction		Family Interaction		Peer Interaction	
	B (SE)	t (*p*)	B (SE)	t (*p*)	B (SE)	t (*p*)	B (SE)	t (*p*)	B (SE)	t (*p*)	B (SE)	t (*p*)
Intercept	21.36 (3.17)	6.73 (<0.001)	3.29 (0.98)	3.37 (0.001)	3.49 (0.994)	3.51 (0.001)	8.58 (0.72)	11.91 (<0.001)	3.12 (0.51)	6.18 (<0.001)	5.46 (0.86)	6.34 (<0.001)
CBM	0.24 (0.05)	4.44 (<0.001)	0.06 (0.02)	3.68 (<0.001)			0.07 (0.02)	4.90 (<0.001)	0.11 (0.01)	9.05 (<0.001)	0.03 (0.02)	2.09 (0.038)
DFC	0.58 (0.10)	5.77 (<0.001)	0.22 (0.04)	5.90 (<0.001)	0.19 (0.03)	6.75 (<0.001)					0.08 (0.03)	2.65 (0.009)
CBF	0.12 (0.05)	2.58 (0.011)			0.05 (0.01)	3.49 (0.001)			0.05 (0.01)	3.87 (<0.001)		
Sex	3.63 (1.36)	2.67 (0.008)			0.87 (0.42)	2.07 (0.040)					0.92 (0.43)	2.15 (0.033)
Religion	−3.22 (1.38)	2.33 (0.020)	−1.21 (0.50)	2.43 (0.016)	−0.94 (0.43)	2.19 (0.030)						
Complications	3.462 (1.59)	2.17 (0.031)							0.89 (0.38)	2.33 (0.020)	1.09 (0.50)	2.18 (0.030)
Disease duration							0.11 (0.03)	3.87 (<0.001)				
adj R^2^	0.420		0.296		0.250		0.128		0.448		0.105	
F (*p*)	30.12 (<0.001)		34.75 (<0.001)		21.13 (<0.001)		18.77 (<0.001)		66.21 (<0.001)		8.09 (<0.001)	
Tolerance	0.576–0.978		0.768–0.957		0.947–0.996		0.998		0.725–0.976		0.780–0.979	
VIF	1.022–1.736		1.045–1.302		1.004–1.056		1.002		1.024–1.379		1.021–1.282	
Durbin-Watson	1.777		2.025		2.117		1.863		1.807		1.944	

Note. PRISM: Problem Recognition in Illness Self-Management, DFCS: Diabetes Family Conflict, CBF: conflict behavior toward father, CBM: conflict behavior toward mother, HbA1C: glycosylated hemoglobin, VIF: variance inflation factor.

## Data Availability

Data sharing not applicable.
